# Scoparone Exerts Anti-Tumor Activity against DU145 Prostate Cancer Cells via Inhibition of STAT3 Activity

**DOI:** 10.1371/journal.pone.0080391

**Published:** 2013-11-15

**Authors:** Jeong-Kook Kim, Joon-Young Kim, Han-Jong Kim, Keun-Gyu Park, Robert A. Harris, Won-Jea Cho, Jae-Tae Lee, In-Kyu Lee

**Affiliations:** 1 Department of Biomedical Science, Graduate School, Kyungpook National University, Daegu, Republic of Korea; 2 Department of Internal Medicine, Research Institute of Aging and Metabolism, World Class University program, Kyungpook National University School of Medicine, Daegu, Republic of Korea; 3 GIST College, Gwangju Institute of Science and Technology, Gwangju, Republic of Korea; 4 Research Institute of Clinical Medicine, Chonnam National University Hwasun Hospital, Jeollanam-do, Republic of Korea; 5 Department of Biochemistry and Molecular Biology, Indiana University School of Medicine, Indianapolis, Indiana, United States of America; 6 College of Pharmacy and Research Institute of Drug Development, Chonnam National University, Gwangju, Republic of Korea; 7 Department of Nuclear Medicine, Kyungpook National University School of Medicine, Daegu, Republic of Korea; University Hospital Carl Gustav Carus Dresden, Germany

## Abstract

Scoparone, a natural compound isolated from *Artemisia capillaris*, has been used in Chinese herbal medicine to treat neonatal jaundice. Signal transducer and activator of transcription 3 (STAT3) contributes to the growth and survival of many human tumors. This study was undertaken to investigate the anti-tumor activity of scoparone against DU145 prostate cancer cells and to determine whether its effects are mediated by inhibition of STAT3 activity. Scoparone inhibited proliferation of DU145 cells via cell cycle arrest in G_1_ phase. Transient transfection assays showed that scoparone repressed both constitutive and IL-6-induced transcriptional activity of STAT3. Western blot and quantitative real-time PCR analyses demonstrated that scoparone suppressed the transcription of STAT3 target genes such as cyclin D_1_, c-Myc, survivin, Bcl-2, and Socs3. Consistent with this, scoparone decreased phosphorylation and nuclear accumulation of STAT3, but did not reduce phosphorylation of janus kinase 2 (JAK2) or Src, the major upstream kinases responsible for STAT3 activation. Moreover, transcriptional activity of a constitutively active mutant of STAT3 (STAT3C) was inhibited by scoparone, but not by AG490, a JAK2 inhibitor. Furthermore, scoparone treatment suppressed anchorage-independent growth in soft agar and tumor growth of DU145 xenografts in nude mice, concomitant with a reduction in STAT3 phosphorylation. Computational modeling suggested that scoparone might bind the SH2 domain of STAT3. Our findings suggest that scoparone elicits an anti-tumor effect against DU145 prostate cancer cells in part through inhibition of STAT3 activity.

## Introduction

Prostate cancer is the second most common cancer and the sixth leading cause of cancer death in men worldwide [1]. Although metastatic prostate cancer responds initially to androgen deprivation therapy, most patients eventually progress to a state of castration-resistant prostate cancer (CRPC) [2,3]. Even with docetaxel-based chemotherapy, treatment of patients with metastatic CRPC remains a major clinical challenge. In this context, the signal transducer and activator of transcription 3 (STAT3) signaling pathway has been validated as a promising therapeutic target in metastatic prostate cancer: this protein is aberrantly activated in prostate cancer and contributes to the promotion of metastatic progression [4,5]. 

STAT3, one of seven STAT family transcription factors, functions as a downstream effector of various cytokines, growth factors, and hormones including IL-6, epidermal growth factor (EGF), and leptin [6-9]. In response to extracellular stimuli, STAT3 is activated through phosphorylation at Y705 and S727 by non-receptor tyrosine kinases, including JAK2 and Src, and by mitogen-activated protein kinases (MAPKs) [10-12]. Phosphorylated STAT3 proteins form dimers that translocate into the nucleus, where they regulate transcription of downstream target genes. Although its phosphorylation is transient and tightly regulated in normal non-transformed cells, STAT3 is persistently activated in a variety of human tumors, including hematological malignancies (leukemia, multiple myeloma and lymphoma) and solid tumors (head and neck squamous cell carcinoma [HNSCC], melanoma, colon, hepatoma, breast, and prostate cancers) [13-17]. A large number of studies have provided convincing evidence for the oncogenic role of constitutively active STAT3 [13-20]. Persistent activation of STAT3 promotes diverse tumorigenic and metastatic processes through transcriptional regulation of various genes involved in cell proliferation (Cyclin D_1_, c-Myc, and p21), survival (Bcl-2, Mcl-1, and Survivin), metastasis (MMP-2 and MMP-9), and angiogenesis (VEGF). Therefore, STAT3 is now considered to represent a promising molecular target for development of anti-cancer therapeutics using both direct and indirect approaches [13,14,21].

Scoparone (6,7-dimethoxycoumarin), a major constituent of the Chinese herb *Artemisia capillaris* (yin chin), has been used for the treatment of neonatal jaundice in Asia [22,23]. The protective effect of scoparone against hyperbilirubinemia is mediated by activating constitutive androstane receptor (CAR), a nuclear hormone receptor that acts as a transcription factor to upregulate expression of enzymes involved in bilirubin clearance. Scoparone also possesses several other biological properties, including anti-coagulant, hypolipidemic, vasorelaxant, anti-oxidant, and anti-inflammatory effects [24-28]. Inhibition of the transcriptional activity of nuclear factor-kappaB (NF-κB) appears responsible for its anti-inflammatory activity [28]. However, the potential anti-tumor activity of scoparone and its molecular targets other than CAR and NF-κB have not been extensively investigated. In this study, we evaluated the anti-tumor activity of scoparone against DU145 androgen-independent prostate-cancer cells and found that its molecular mechanisms of action are associated with inhibition of STAT3 activity.

## Materials and Methods

### Reagents and plasmid constructs

Scoparone (6,7-dimethoxycoumarin), AG490 (a JAK2 inhibitor), TNF-α, forskolin (FSK), and phorbol 12-myristate 13-acetate (PMA) were purchased from Sigma-Aldrich (St. Louis, MO, USA). IL-6 was obtained from BD Biosciences (San Jose, CA, USA). The M67-Luc reporter construct and the expression vectors for wild-type STAT3 or a constitutively active form of STAT3 (STAT3C) [20] were kind gifts from Dr. James E. Darnell (The Rockefeller University, New York, NY, USA). Reporter constructs (NF-κB-Luc, AP-1-Luc, CRE-Luc, and Egr-1-Luc) and expression vector for Egr-1 were described previously [29]. The pTOPFLASH luciferase reporter construct [30] and the expression vector for dominant active mutant of human β-catenin (ΔN-β-catenin) containing an in-frame N-terminal deletion of amino acids 29−48 [31] were kindly donated by Dr. Hans Clevers (University Medical Center Utrecht, Utrecht, Netherlands) and Dr. Frank McCormick (University of California, San Francisco, CA, USA), respectively. To generate the Vxy Puro-Luc construct, cDNA encoding firefly luciferase was amplified by PCR and inserted into the *Bam*H1*/Xho*1 sites of plasmid Vxy-puro [12]. 

### Ethics statement

All experimental procedures were conducted in strict accordance with the appropriate institutional guidelines for animal research. The protocol was approved by the Committee on the Ethics of Animal Experiments of the Kyungpook National University (Permit Number: 2013-0015). 

### Cell culture and transient transfection assay

Human prostate cancer cell lines (DU145 and PC-3), an immortalized human prostate epithelial cell line (RWPE-1), human breast cancer cell lines (MCF-7 and MDA-MB-231), human hepatocellular carcinoma cell lines (HepG2 and Hep3B), a cervical cancer cell line (HeLa), and colon cancer cell lines (HT-29, HCT-116, and HCT-15) were obtained from the American Type Culture Collection (ATCC; Manassas, VA, USA). DU145, PC-3, MDA-MB-231, HeLa, HT-29, HCT-116, and HCT-15 cells were grown in RPMI (Invitrogen, Carlsbad, CA, USA) medium supplemented with 10% fetal bovine serum (FBS; Hyclone, Logan, UT, USA) and antibiotics. MCF-7 and Hep3B cells were grown in DMEM (Invitrogen) medium supplemented with 10% FBS and antibiotics. RWPE-1 cells were grown in complete Keratinocyte serum-free medium (K-SFM; Invitrogen) containing 50 μg/mL bovine pituitary extract, 5 ng/mL human recombinant EGF, and antibiotics. HepG2 cells were cultured in MEM (Invitrogen) supplemented with 10% FBS and antibiotics. For transient transfection assays, HepG2 (8 × 10^4^), DU145 (3 × 10^4^), and PC-3 (3 × 10^4^) cells were seeded in 24-well plates and transfected with the M67-Luc reporter construct (200 ng/well), with or without expression vector (100 ng/well) for either wild-type or constitutively active mutant STAT3 (STAT3C) using the TransIT-LT1 transfection reagent (Mirus Bio LLC, Madison, WI, USA). HepG2 cells were also transfected with pTOPFLASH or Egr-1-Luc reporter plasmids (200 ng/well) together with or without expression plasmids (100 ng/well) for ΔN-β-catenin or Egr-1, respectively. pSV40-β-galactosidase expression plasmid was used as an internal control. At 24 h after transfection, cells were stimulated with IL-6 (10 ng/mL) for 24 h if required, and then harvested for luciferase and β-galactosidase assays. Luciferase activity was normalized against β-galactosidase activity. 

### Cell proliferation assay

Cell proliferation assays were performed using the WST-8 cell proliferation assay kit, as previously described [29]. Cells were seeded in 96-well plates at a density of 1.5 × 10^3^ cells/well and serum starved for 24 h. Cells were then stimulated with 10% FBS in the presence of 0.1% DMSO (vehicle) or 0.1, 1, 10, 50, 100, 200, 500 or 1000 μmol/L of scoparone for 72 h. Cells were then incubated at 37°C for further 2 h in medium containing WST-8 reagent (Dojindo Laboratories, Kumamoto, Japan). The absorbance at 450 nm was measured to determine cell proliferation. 

### Flow cytometric analysis of the cell cycle

Flow cytometric cell cycle analysis was carried out as previously described [29]. Briefly, DU145 cells (4 × 10^5^ cells/100 mm culture dish) were synchronized in G_0_/G_1_ phase by serum starvation for 24 h. Based on a previous report [32] showing that serum-starved DU145 cells progressed from G_1_ to S phase of the cell cycle after 27.5 h of serum stimulation, cells were stimulated with 10% FBS in the presence of 0.1% DMSO or scoparone (0.5 and 1 mmol/L) for 28 h. Cells were harvested, and then analyzed with a FACSCalibur flow cytometer (BD Bioscience). 

### Western blot analysis

Western blot analysis was performed as previously described with minor modifications [12,29]. Proteins were resolved by SDS-PAGE and transferred to Immobilon-P-membrane (Millipore, Billerica, MA). After blocking, the membrane was incubated with primary antibodies against Cyclin D_1_, phospho-pRb (ppRb), JAK2, phospho-JAK2 Tyr1007/1008 (pJAK2), Src, phospho-Src family Tyr416 (pSrc), STAT3, phospho-STAT3 Tyr705 (pSTAT3 Y705), phospho-STAT3 Ser727 (pSTAT3 S727), Survivin, c-Myc, SOCS3, and Bcl-2 (Cell Signaling, Beverly, MA, USA); Cyclin E (Santa Cruz Biotechnology, Santa Cruz, CA, USA), and β-actin (Sigma-Aldrich). After washing, the membranes were incubated with horseradish peroxidase (HRP)- or IRDye-conjugated (IRDye 800CW and IRDye 680LT, LI-COR Biosciences, Lincoln, NE) secondary antibodies, and signals were detected using the ECL Western blot detection system (Amersham, Buckinghamshire, UK) or Odyssey Infrared Imaging System (LI-COR Biosciences, Lincoln, NE, USA), respectively. β-actin was used as the protein loading control. Signal intensities were quantified by densitometry using the Image J software and normalized against the corresponding β-actin signals.

### Quantitative real-time PCR (qRT-PCR) analysis

DU145 cells were incubated with scoparone for 1, 3, 6, 12, or 24 h. Total RNA was extracted using the QIAzol lysis reagent (Qiagen, Valencia, CA, USA), and 1 µg of total RNA was reverse transcribed using the RevertAid First Strand cDNA synthesis kit (Thermo Scientific, Waltham, MA, USA) according to the manufacturers’ instructions. For qRT-PCR, 25−50 ng of cDNA was used for PCR amplification (annealing temperature : 60C) using Power SYBR Green PCR Master Mix (Applied Biosystems, Warrington, UK) with the ViiA™ 7 Real-Time PCR System (Applied Biosystems). Acidic ribosomal phosphoprotein P0 (RPLP0; 36B4) was used as an internal control. PCR condition and primer sequences are listed in Table S1.

### Immunofluorescence analysis

DU145 cells were seeded on glass coverslips and allowed to attach for 24 h. Based on our Western blot results showing sustained reduction in STAT3 phosphorylation from 2−6 h after scoparone treatment, cells were treated with scoparone (0.5 mmol/L) for 4 h. Cells were fixed with 95% ethanol for 15 min at −20°C, and then incubated with antibodies against pSTAT3 (Y705) or STAT3 followed by incubation with Alexa Fluor 488- or Alexa Fluor 568-labeled secondary antibodies, respectively. Cell nuclei were stained with DAPI and confocal images were obtained and merged.

### Anchorage-independent growth assay

Soft agar colony-formation assays were performed as previously described with minor modifications [12]. DU145 (1 × 10^4^) cells in 1.5 mL of growth medium were mixed with 1.5 mL of 0.5% agarose in warmed growth medium containing vehicle (0.1% DMSO) or 50, 100, or 200 μmol/L of scoparone and layered on 0.5% base agar in 60-mm cell culture dishes. Culture medium containing scoparone was added only once; subsequently, medium without scoparone was added every week for 21 days until large colonies were evident. Cells were stained with crystal violet for colony counting.

### Molecular modeling and docking study

The molecular modeling and docking study was performed using Surflex-Dock in SYBYL version 8.1.1 by Tripos Associates (St. Louis, MO, USA), as previously described [33]. The crystal structure of STAT3 was retrieved from the Protein Data Bank (PDB ID code 1BG1) and refined for the docking process [34]. The structure of scoparone was created using the SYBYL package with standard bond lengths and angles, and minimized using the conjugate gradient method until the gradient was 0.001 kcal/mol with the Tripos force field. The Gasteiger-Huckel charge, with a distance-dependent dielectric function, was applied for the minimization process. The image of the predicted interaction was created with the molecular graphics program MOLCAD. After running Surflex-Dock, the conformer with best total score was selected and used to predict the detailed binding patterns in the cavity.

### Establishment of stable cell lines expressing firefly luciferase

To generate retroviruses expressing firefly luciferase, Phoenix A cells were transfected with the Vxy Puro-Luc construct as described previously [12]. DU145 cells were transduced with retroviruses expressing firefly luciferase and selected with puromycin (1 μg/mL) for 2 weeks. Selected DU145 (DU145-Luc) cells were verified for luciferase activity and for the anti-proliferative effect of scoparone (unpublished data). 

### Xenograft tumor model and immunohistochemical analyses

Six-week-old athymic male BALB/c nude (nu/nu) mice (Japan SLC, Inc., Shizuoka, Japan) were acclimatized under controlled standard conditions for a week prior to the experiment according to Guide for the Care and Use of Laboratory Animals of the National Institute of Health. DU145-Luc (5 × 10^6^) cells in 100 μL of PBS were subcutaneously injected into the right dorsal flank area of male athymic nude mice (BALB/c Slc-*nu/nu*, 7 weeks old). The diameter of the tumor was measured externally with a caliper ruler every 2 days, and the volume of the tumor (mm^3^) was estimated. After 7 days, when tumors reached an average size of approximately 20−25 mm^3^, mice were imaged on the IVIS imaging system (Caliper Life Sciences, Hopkinton, MA, USA) 10 min after i.p. injection of 100 μL XenoLight D-luciferin (30 mg/mL, Caliper). The animals were then randomly divided into two groups (n=8/group) and given intraperitoneal (i.p.) injections of either vehicle or scoparone (30 mg/kg) every 2−3 days for 18 days. Mice were anesthetized with sodium pentobarbital (Entobar; Hanlim Pharmaceutical, Yong-In, Korea) and imaged on the IVIS imaging system. Xenograft tumors were excised for volume measurement and immunohistochemistry. For immunohistochemical analysis, tumor tissues were removed, formalin-fixed and paraffin-embedded. Serial 4-μm tumor sections were deparaffinized in xylene, rehydrated through descending grades of ethanol, and either stained with hematoxylin and eosin (H&E) or subjected to immunohistochemical analysis using antibodies against pSTAT3 (Y705) and Survivin as previously described [29].

### Statistical analysis

Data are expressed as means ± S.E.M. Statistical analyses were performed with the unpaired Student’s *t*-test, and a value of *P* < 0.05 was considered statistically significant.

## Results

### Scoparone elicits an anti-proliferative effect on DU145 human prostate cancer cells by inducing cell-cycle arrest in G_1_ phase

**Figure 1:**
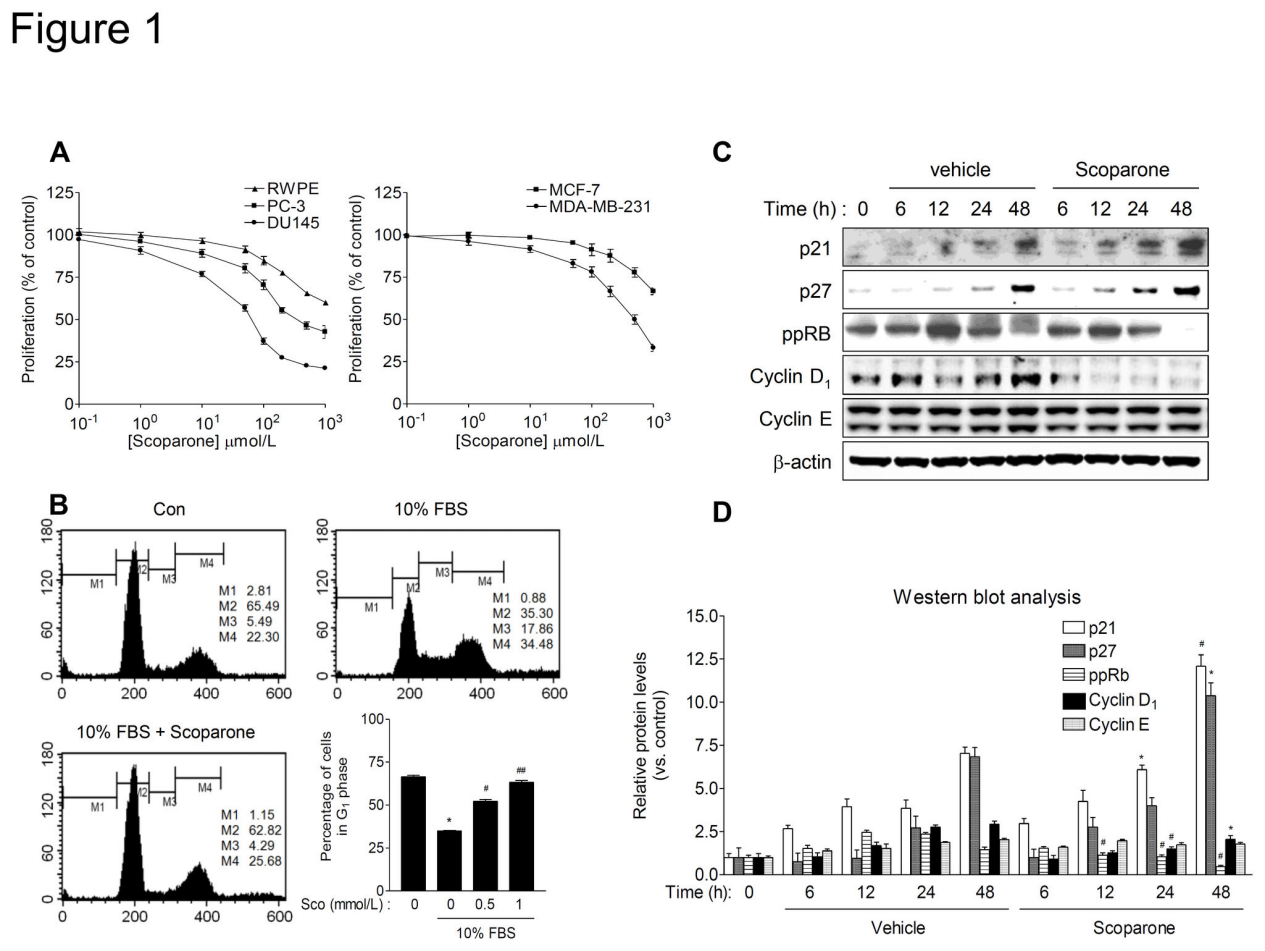
Scoparone suppresses proliferation of DU145 prostate cancer cells by inducing G_1_-phase cell-cycle arrest. A and B. Scoparone inhibits proliferation of human cancer cell lines. Prostate cancer cells (DU145 and PC-3), RWPE-1 (an immortalized human prostate epithelial cell line), and breast cancer cells (MCF-7 and MDA-MB-231) were serum starved for 24 h. Cells were then incubated in growth medium supplemented with 10% FBS in the presence of vehicle (0.1% DMSO) or the indicated concentrations of scoparone for 72 h, and cell proliferation was determined by WST-8 assay. Data are expressed as percentages of the vehicle control (defined as 100%) and represent the means ± S.E.M. of three independent experiments, each performed in triplicate. B. Scoparone induces cell-cycle arrest at G_1_ phase in DU145 cells. Serum-starved DU145 cells were stimulated with 10% FBS in the presence of vehicle or scoparone (0.5 and 1 mmol/L) for 28 h, and then subjected to flow cytometric analysis of the cell cycle. Bar graph indicates the percentage of cells in G_1_ phase. The data are the means ± S.E.M. of three independent experiments, each performed in duplicate. ^*^ *P* < 0.001 vs. vehicle control; ^#^ *P* < 0.005, ^##^ *P* < 0.001 vs. serum stimulation. C. Representative Western blot analysis of cell cycle-related proteins. DU145 cells were treated with scoparone (0.5 mmol/L) for the indicated times, and then harvested for Western blot analysis. D. Quantification of relative protein levels. Signal intensities were quantified by densitometric analysis and normalized to that of the corresponding β-actin signal. Expression level of each protein was expressed as a ratio relative to the level in the control at time 0 h, which was defined as 1. The data represent the means ± S.E.M of three independent experiments. ^*^ *P* < 0.05, ^#^ *P* < 0.005 vs. vehicle control at each time point.

To evaluate the anti-cancer potential of scoparone on prostate cancer cells *in vitro*, we performed a WST-8 cell proliferation assay on two androgen-independent human prostate cancer cell lines (DU145 and PC-3) and an immortalized human prostate epithelial cell line (RWPE-1). Scoparone treatment for 72 h significantly inhibited the proliferation of DU145 cells, with an IC_50_ value of 41.3 μmol/L. However, the drug exerted little growth-inhibitory effect on PC-3 and RWPE-3 cells at that concentration (Figure 1A). Nevertheless, both cell lines showed reduced proliferation at higher concentrations of the drug (> 100 μmol/L), which was also observed for several other cell lines derived from breast cancers (MCF-7 and MDA-MB-231), hepatocellular carcinomas (HepG2 and Hep3B), cervical cancer (HeLa), and colon cancers (HCT-15 HCT-116 and HT-29) (Figure 1A and Figure S1), suggesting that scoparone has a general anti-proliferative effect on cancer cells. 

To further determine whether the anti-proliferative effect of scoparone is due to inhibition of cell-cycle progression or induction of apoptosis, we carried out a flow cytometric analysis of the cell cycle in DU14 cells, which were the most sensitive to the growth-inhibitory effect of scoparone. Consistent with a previous report [32], the percentage of DU145 cells progressing into the S phase of the cell cycle was significantly increased after 28 h of serum stimulation. In the presence of scoparone, the serum-stimulated increase in the S-phase cell population was reduced, and the number of cells in G_1_ phase was concurrently increased (Figure 1B). However, the sub G_1_ peak, indicative of an apoptotic cell population was not increased by scoparone. Thus, in DU145 cells, scoparone induces cell-cycle arrest at G_1_ phase without induction of apoptosis. 

The G_1_-phase cell-cycle arrest induced by scoparone was further confirmed by examining the cellular levels of cell-cycle regulatory proteins that promote the G_1_-S transition. Western blot analyses revealed that scoparone increased the levels of cell cycle inhibitory proteins such as p21 and p27, which are inhibitors of the Cyclin/Cdk complex (Figure 1C and D). By contrast, it significantly reduced the levels of the cell-cycle promoting proteins Cyclin D_1_ and phosphorylated pRb (ppRb), although it had little effect on the expression of Cyclin E. These findings demonstrate that scoparone induces G_1_-phase cell-cycle arrest by altering the levels of proteins that influence cell-cycle progression.

### Scoparone inhibits constitutive and IL-6-stimulated transcriptional activity of STAT3

**Figure 2:**
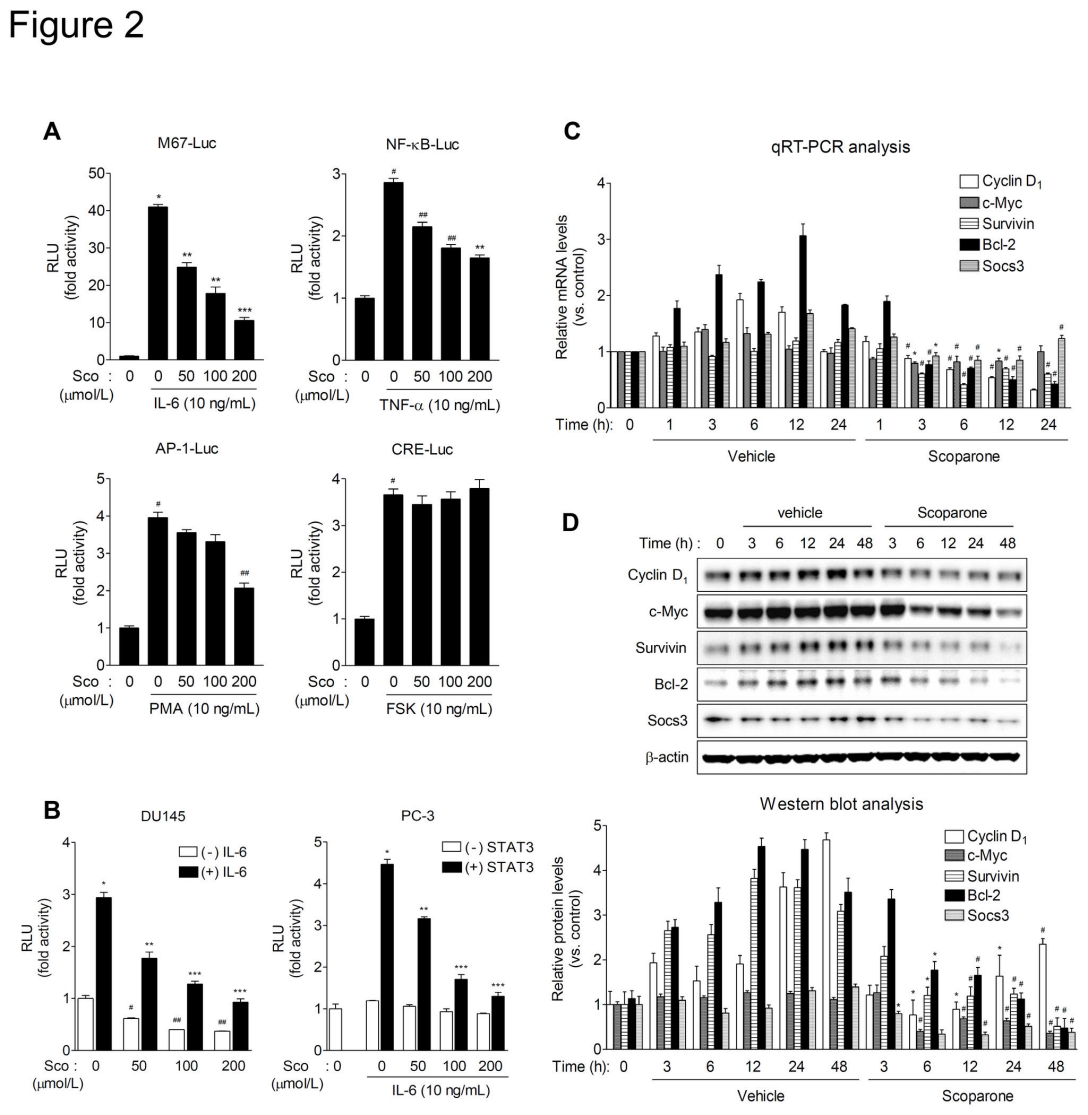
Scoparone inhibits constitutive and IL-6-stimulated transcriptional activity of STAT3. A. The effect of scoparone on the activities of transcriptional factors. HepG2 cells were transiently cotransfected with various luciferase reporter plasmids driven by synthetic promoters containing binding sites for relevant transcription factors. At 24 h after transfection, cells were treated with the appropriate upstream stimulators and scoparone for 24 h, and then harvested for luciferase and β-galactosidase assays. RLU, relative luminescence units. Data are the means ± S.E.M. of three independent experiments, each performed in duplicate. ^*^ *P* < 0.0005, ^#^ *P* < 0.005 vs. reporter alone; ^**^ *P* < 0.005, ^***^ *P* < 0.001, ^##^ *P* < 0.01 vs. stimulation. B. Scoparone inhibits both constitutive and IL-6-stimulated transcriptional activity of STAT3. DU145 cells were transiently transfected with M67-Luc, a synthetic promoter containing four copies of the STAT3 binding site. PC-3 cells were co-transfected with M67-Luc construct and the expression vector for wild-type STAT3. At 24 h after transfection, cells were treated with IL-6 in the presence of scoparone for 24 h. Data are the means ± S.E.M. of three independent experiments, each performed in duplicate. ^*^ *P* < 0.005, ^#^ *P* < 0.05, ^##^ *P* < 0.01 vs. reporter alone; ^**^ *P* < 0.01, ^***^ *P* < 0.005 vs. IL-6 stimulation. C and D. qRT-PCR and Western blot analyses of STAT3 downstream target genes. DU145 cells were treated with scoparone (0.5 mmol/L) for the indicated times, and then harvested for qRT-PCR (C) and Western blot (D) analyses. mRNA levels of STAT3 target genes were determined by qRT-PCR analysis (C) and normalized against the level of RPLP0/36B4. Data are expressed as means ± S.E.M. of three independent experiments, each performed in triplicate. ^*^ *P* < 0.05, ^#^ *P* < 0.005 vs. vehicle control at each time point. Protein expression levels (D) were quantified by densitometric analysis and normalized against the corresponding levels of β-actin. Data are expressed as means ± S.E.M. of three independent experiments. ^*^ *P* < 0.05, ^#^ *P* < 0.005 vs. vehicle control at each time point.

To identify transcription factors that are targeted by scoparone, we assessed its effect on the transcriptional activity of several transcription factors that play important roles in cancer cell proliferation. Interestingly, scoparone strongly suppressed IL-6-stimulated STAT3 transcriptional activity (Figure 2A) and, as reported, repressed TNF-α-stimulated NF-κB transcriptional activity. Scoparone also slightly inhibited PMA-induced AP-1 activity and Egr-1 activity, whereas it exerted little effect on either CREB- or β-catenin-dependent transcription (Figure S2). 

To validate the inhibitory effect of scoparone on the transcriptional activity of STAT3, we performed transient transfection assays using DU145 cells expressing constitutively active STAT3 and PC-3 cells lacking STAT3 [35]. Scoparone significantly inhibited constitutive and IL-6-enhanced transcriptional activity of STAT3 in DU145 cells (Figure 2B, left). In STAT3-negative PC-3 cells, as expected, basal luciferase activity was extremely low; furthermore, IL-6 treatment did not increase reporter gene activity, which was not affected by scoparone (Figure 2B, right). However, overexpression of wild-type STAT3 led to increase in luciferase activity in response to IL-6 stimulation of PC-3 cells. Scoparone treatment considerably reduced the IL-6-stimulated STAT3 activity. These results suggest that scoparone inhibits both constitutive and IL-6-stimulated transcriptional activity of STAT3. 

To further investigate whether scoparone can repress the transcription of STAT3 downstream targets, we performed quantitative real-time PCR (qRT-PCR) analyses for STAT3 target genes such as Cyclin D_1_, c-Myc, Survivin, Bcl-2, and Socs3. qRT-PCR analyses demonstrated that scoparone decreased the mRNA expression levels of the STAT3 target genes (Figure 2C). In agreement with qRT-PCR results, it also significantly diminished the protein levels of those genes (Figure 2D). These findings demonstrate that scoparone suppresses of STAT3 target gene transcription. 

### Scoparone inhibits phosphorylation and nuclear accumulation of STAT3 independently of JAK2 and Src

**Figure 3:**
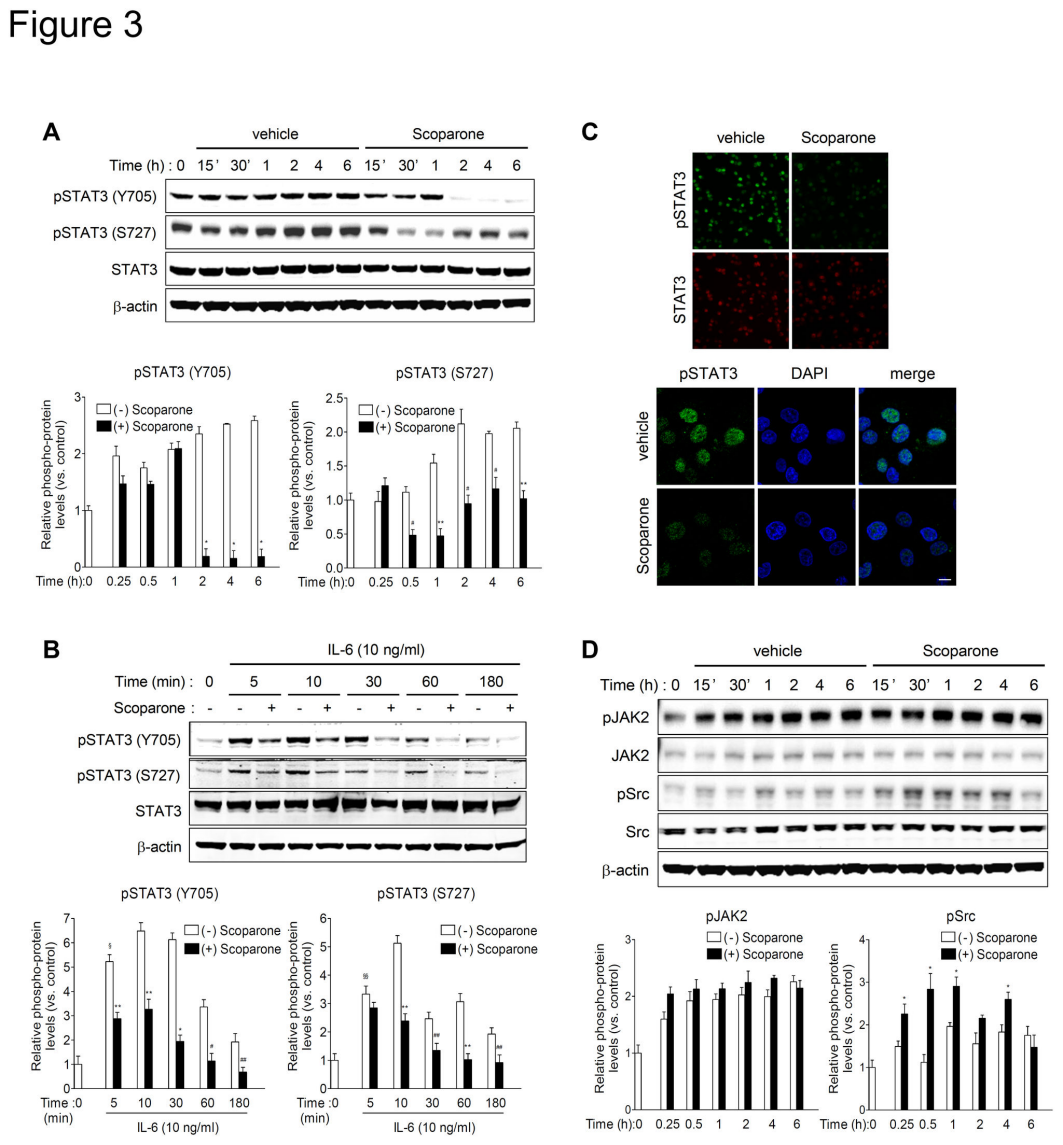
Scoparone inhibits phosphorylation and nuclear localization of STAT3 independently of JAK2 or Src in DU145 cells. A and B. Scoparone inhibits both constitutive (A) and IL-6-enhanced (B) phosphorylation of STAT3 at Tyr705 and Ser727. DU145 cells were treated with vehicle or scoparone (A), or serum-starved for 24 h followed by scoparone treatment (0.5 mmol/L) for 4 h prior to stimulation with IL-6 (B) for the indicated times. Cells were harvested for Western blotting using antibodies against pSTAT3 (Y705), pSTAT3 (S727), and STAT3. Protein levels were quantified by densitometry and normalized against the corresponding levels of β-actin. Expression level of each protein iss expressed as a ratio relative to the level in the control at time 0 h (defined as 1). The data represent the means ± S.E.M of three independent experiments. ^§^ *P* < 0.0005 and ^§§^ *P* < 0.005 vs. control at time 0; ^*^ *P* < 0.0005, ^**^ *P* < 0.005, ^#^ *P* < 0.01 ^##^ *P* < 0.05 vs. vehicle control at each time point. C. Scoparone reduces nuclear accumulation of phosphorylated STAT3. DU145 cells were treated with scoparone for 4 h and processed for immunofluorescence staining with antibodies against pSTAT3 (Y705) or STAT3. Cell nuclei were stained with DAPI, and confocal images were obtained and merged. Scale bar indicates 10 μm. D. Scoparone did not inhibit phosphorylation of JAK2 and Src, two major STAT3 upstream kinases. DU145 cells were treated with scoparone for the indicated times, and then harvested for Western blot analysis. Signal intensities were quantified by densitometric analysis and normalized against the corresponding β-actin signals. Expression level of each protein is expressed as a ratio relative to the level in the control at time 0 h, which was defined as 1. ^*^ *P* < 0.05 vs. vehicle control at each time point.

To delineate the molecular mechanisms underlying inhibition of STAT3 activity by scoparone, we evaluated its effect on STAT3 phosphorylation in DU145 cells. Scoparone significantly reduced the levels of STAT3 phosphorylation at Tyr705 (pSTAT3 Y705) and Ser727 (pSTAT3 S727) in DU145 cells 2 h and 30 min after treatment, respectively (Figure 3A). It also decreased IL-6-enhanced phosphorylation of STAT3 (Figure 3B). Total protein levels of STAT3 were not affected by scoparone treatment (Figure 3A and B, and Figure S3). Consistent with these findings, immunofluorescence staining of pSTAT3 Y705 showed that pSTAT3 proteins were predominantly localized in the nucleus of DU145 cells (Figure 3C). The nuclear pSTAT3 signal was markedly reduced in scoparone-treated cells. These results demonstrate that scoparone inhibits constitutive and IL-6-stimulated phosphorylation and nuclear accumulation of pSTAT3 in DU145 cells.

To determine whether the inhibitory effect of scoparone on STAT3 phosphorylation is due to the suppression of upstream signaling events, we assessed the effect of scoparone on phosphorylation of JAK2 and Src, two major upstream kinases responsible for STAT3 activation. Scoparone did not reduce the protein levels of constitutively phosphorylated JAK2 (Figure 3D) and Src phosphorylation was not reduced, but rather slightly elevated, by scoparone. The total protein and mRNA levels for JAK2 and Src were not altered by scoparone treatment (Figure 3D and Figure S4). Collectively, these observations suggest that scoparone inhibits phosphorylation and nuclear accumulation of STAT3 independently of the upstream kinases JAK2 or Src. 

### Scoparone may inhibit transcriptional activity of STAT3 by direct binding to the SH2 domain of STAT3

**Figure 4:**
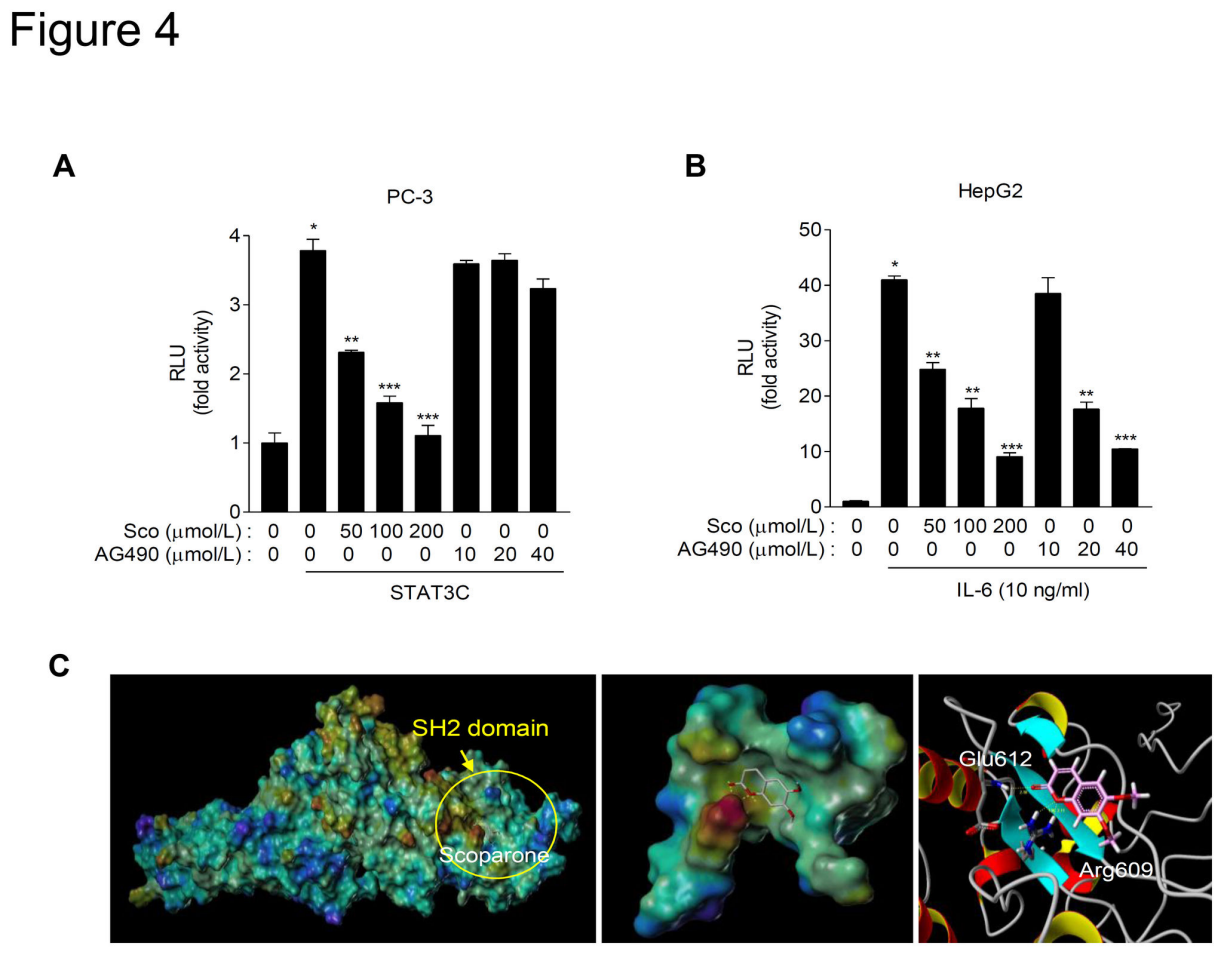
Scoparone may inhibit transcriptional activity of STAT3C by binding the SH2 domain of STAT3. A. Scoparone, but not the JAK2 inhibitor AG490, suppresses transcriptional activity of STAT3C, a constitutive active form of STAT3. PC-3 cells were transiently transfected with M67-Luc and expression plasmids for STAT3C. After 24 h, cells were treated with AG490 or scoparone for 24 h. Data are the means ± S.E.M. of three independent experiments, each performed in duplicate. ^*^ *P* < 0.005 vs. reporter alone; ^**^ *P* < 0.01, ^***^ *P* < 0.005 vs. STAT3C co-transfection. B. Both scoparone and AG490 inhibited IL-6-stimulated STAT3 activity. HepG2 cells were transiently transfected with M67-Luc for 24 h, and then stimulated with IL-6 in the presence of either AG490 or scoparone for an additional 24 h. Data are the means ± S.E.M. of three independent experiments, each performed in duplicate. ^*^ *P* < 0.0005 vs. reporter alone; ^**^ *P* < 0.005, ^***^ *P* < 0.0005 vs. IL-6 stimulation. C. Scoparone may directly bind to the SH2 domain of STAT3. *a*, docking simulation of scoparone binding to STAT3 using Surflex-Dock computational modeling. The molecular surface of STAT3 is colored based on its electrostatic potential (blue, positive; red, negative). *b*, scoparone docked to the SH2 domain of STAT3. Oxygen atoms of scoparone are shown in red, and hydrogen bonds formed between scoparone and SH2 domain of STAT3 are shown by white dashed lines. *c*, detailed binding mode of scoparone to amino acid residues within the SH2 domain of STAT3. The carbonyl oxygen and the adjacent oxygen atoms of lactone of scoparone form hydrogen bonds with Glu612 and Arg609 with 2.18 Å and 2.11 Å distances, respectively.

To further confirm whether scoparone could regulate STAT3 activity independently of upstream signaling, PC-3 cells were transiently transfected with STAT3C, a constitutively active mutant of STAT3 that mimics the action of phosphorylated STAT3 by substitution of two cysteine residues within its SH2 domain and activates target gene transcription without upstream stimuli [20]. Overexpression of STAT3C was sufficient to increase M67-Luc activity without IL-6 stimulation in PC-3 cells (Figure 4A). Intriguingly, the transcriptional activity of STAT3C was remarkably inhibited by scoparone, but not by AG490, an inhibitor of JAK2. However, AG490 efficiently suppressed that of wild-type STAT3 (Figure 4B). These results imply that unlike AG490, scoparone may inhibit constitutive transcriptional activity of STAT3 independently of upstream signaling. 

Because scoparone inhibits STAT3C activity, we postulated that scoparone might regulate STAT3 activity possibly via direct binding to STAT3. To explore this possibility, we carried out a structure-based molecular modeling and docking study. The refined model suggested that two adjacent oxygen atoms within the lactone ring of scoparone might form hydrogen bonds with nearby amino acid residues, Glu612 and Arg609 within the SH2 domain of STAT3, at distances of 2.18 Å and 2.11 Å, respectively (Figure 4C). These results suggest that scoparone inhibits the constitutive transcriptional activity by binding to the SH2 domain of STAT3. 

### Scoparone inhibits anchorage-independent cell growth *in vitro* and xenograft tumor growth of DU145 cells in nude mice

**Figure 5:**
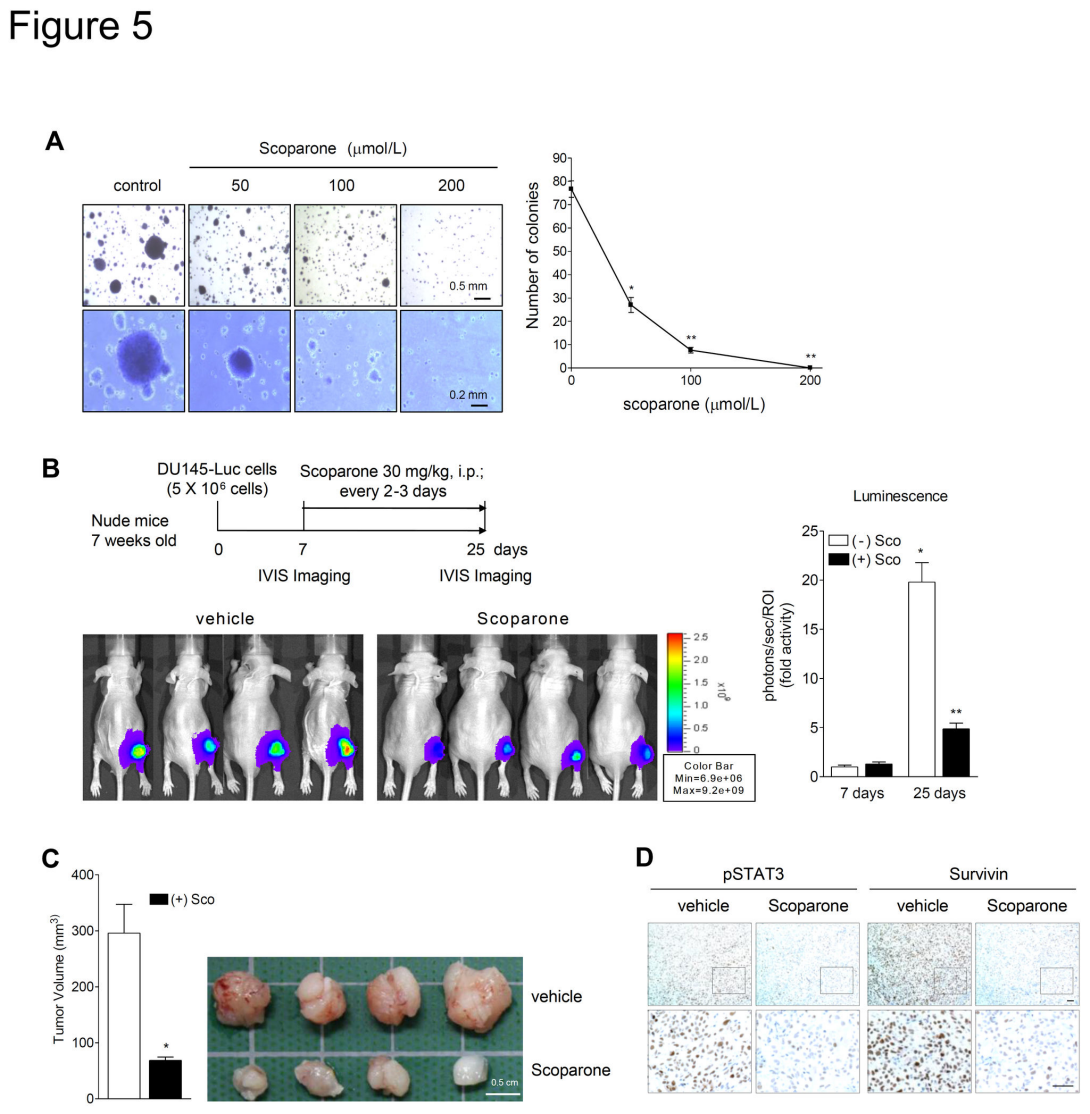
Scoparone suppresses anchorage-independent growth *in* *vitro* and xenograft tumor growth of DU145 cells in nude mice. A. Scoparone inhibits anchorage-independent growth of DU145 cells. DU145 cells were grown for 3 weeks in 0.25% agarose gel containing vehicle or scoparone. The number of colonies lager than 2 mm in diameter was counted and data represent the means ± S.E.M. of three independent experiments, each performed in duplicate. ^*^ *P* < 0.0005, ^*^ *P* < 0.00005 vs. vehicle. B and C. *In* *vivo* anti-tumor effect of scoparone against DU145 xenografts in nude mice. DU145-Luc cells were injected subcutaneously into right hind limbs of athymic nude mice. Seven days after xenograft implantation, mice were divided into two groups and given vehicle or scoparone for 18 days. The animals were subjected to *in* *vivo* bioluminescence imaging (B) and ultimately sacrificed to measure the tumor volume (C). The graphs indicate bioluminescence intensity (B, right panel) and tumor volume (C, left panel). Data are the means ± S.E.M, n=6 mice in each group. ^*^ *P* < 0.005 vs. 7 days after xenograft implantation, ^**^ *P* < 0.005 vs. vehicle-treated group at 25 days (B), ^*^ *P* < 0.005 vs. vehicle-treated group (C). D. Scoparone decreased STAT3 phosphorylation and Survivin expression in DU145 xenografts. Xenograft tumor sections were subjected to immunohistochemical analysis for pSTAT3 and Survivin. Scale bars represent 50 μm.

To verify whether scoparone could inhibit anchorage-independent growth of DU145 cells, we performed soft agar colony formation assays. Scoparone greatly decreased, in a dose-dependent manner, the number and the size of colonies of DU145 cells grown in soft agar. (Figure 5A), suggesting that scoparone inhibits the *in vitro* transformation capacity of DU145 cells. 

To confirm the anti-tumor effect of scoparone *in vivo*, we performed a xenograft assay of DU145 cells in athymic nude mice. For *in vivo* imaging, we used retroviral transduction to establish DU145-Luc cells that stably express firefly luciferase. As shown in Figure 5B, the intensities of luciferase images revealed that the growth of DU145 xenografts was significantly reduced in the scoparone-treated group, compared with that of the vehicle-treated group. Additionally, scoparone treatment markedly reduced the tumor volume of DU145 xenografts (Figure 5C). 

To ascertain whether scoparone inhibits STAT3 phosphorylation and function in DU145 xenografts in nude mice, we performed immunohistochemistry (IHC) for pSTAT3 (Y705) and Survivin. Immunohistochemical analyses revealed that scoparone treatment significantly decreased signals of pSTAT3 and Survivin (Figure 5D). These results demonstrated that scoparone suppresses the transforming capacity of DU145 *in vitro* and exerts an anti-tumor effect against DU145 xenografts *in vivo*, at least in part through inhibition of STAT3.

## Discussion

In this study, we demonstrated the anti-tumor effects of scoparone on DU145 prostate cancer cells both *in vitro* and *in vivo*, and partially delineated the underlying molecular mechanism. Scoparone inhibited transcriptional activity of STAT3 and thereby suppressed transcription of oncogenic STAT3 target genes, leading to growth inhibition of DU145 cells. *In silico* docking studies and inhibition of STAT3C activity by scoparone suggested that STAT3 might be a direct molecular target of scoparone. We also verified that scoparone efficiently suppressed anchorage-independent growth in soft agar and xenograft tumor growth of DU145 cells in nude mice. Thus, our findings suggest that scoparone acts at least partly through inhibition of constitutively activated STAT3, and that it represents a novel candidate for a chemotherapeutic agent against prostate cancer.

Scoparone, a coumarin derivative, is a phytochemical derived from the traditional Chinese herb Yin Chin. The drug has multiple beneficial effects, promoting bilirubin clearance and exhibiting anti-inflammatory, hypolipidemic, anti-coagulant, and anti-oxidant activities [22-28]. Some of its biological activities are mediated in part by activating CAR or inhibiting NF-κB [23,28]. However, its various biological properties cannot be totally explained by regulation of these transcription factors, and its anti-tumor potential remains to be determined. In this study, scoparone inhibited proliferation and anchorage-independent growth of DU145 prostate cancer cells *in vitro* and xenograft tumor growth in nude mice. Furthermore, we identified STAT3 as a novel molecular target of scoparone. Scoparone inhibits phosphorylation, nuclear accumulation and subsequent transcriptional activity of STAT3, probably by direct binding to the SH2 domain of STAT3. This leads to repression of STAT3 target genes (Cyclin D_1_, c-Myc, Survivin, and Bcl-2) that are involved in cell proliferation and survival. Consistent with these findings, scoparone suppressed the proliferation of DU145 cells with constitutively active STAT3 more potently than that of STAT3-negative PC-3 cells. In addition, scoparone inhibited proliferation of hepatoma, cervical cancer and colon cancer cell lines that harbor constitutively active STAT3. Therefore, our study suggests that STAT3 is a novel molecular target for scoparone, and that scoparone represents an anti-cancer drug candidate for the treatment of cancers in which the STAT3 signaling pathway is constitutively active. 

Given that there are no known naturally occurring activating mutations in the *STAT3* gene, aberrant activation of STAT3 in many types of cancers is primarily due to overexpression or deregulation of upstream signaling molecules such as IL-6/gp130, EGF/EGFR, JAK2, or Src [36-38]. For example, prostate cancer cells express high levels of IL-6, EGF, and EGFR, leading to constitutive activation of JAK2 or Src, which in turn results in STAT3 activation through autocrine and paracrine mechanisms [39,40]. Regardless of which upstream signaling molecules are deregulated, the tumor-promoting effects of persistent activation of these signaling pathways are ultimately mediated by STAT3-dependent transcriptional regulation of downstream proto-oncogenes. Therefore, direct or indirect inhibition of STAT3 activity has been suggested as a novel anti-cancer therapeutic strategy [13,14,21,41-44]. In this study, we found that scoparone suppressed STAT3 phosphorylation independently of upstream kinases JAK2 and Src, and that scoparone but not AG490 inhibited transcriptional activity of a constitutively active form of STAT3 (STAT3C). Consistent with these findings, computational modeling proposed that two oxygen atoms in the lactone of scoparone might form hydrogen bonds with Glu612 and Arg609 within the SH2 domain of STAT3. These two residues are considered critical for the interaction with the phosphorylated tyrosine when STAT3 proteins dimerize or are recruited to docking sites within EGFR or gp130 to be phosphorylated [44]. Therefore, scoparone may affect dimerization, phosphorylation, and subsequent nuclear localization of STAT3, possibly via binding to the SH2 domain of STAT3 protein. This mechanism is in contrast to the indirect activation of CAR and inhibition of NF-κB [23,28] by scoparone. However, further studies are needed to conclusively demonstrate that scoparone directly binds to the SH2 domain of STAT3.

Over 70% of anti-cancer drugs developed in the last three decades are natural products, derived from plants, animals, or microorganisms, or natural product-derived substances [45-47]. Etoposide and paclitaxel (taxol) are phytochemicals that are currently used as anti-cancer drugs. However, due to their non-specific cytotoxicity and adverse side effects, many ongoing studies aim to identify natural products that have greater potency with fewer side effects than conventional anti-cancer drugs. Besides, combining natural compounds with chemotherapeutic drugs is an attractive strategy for augmenting inhibition of tumor growth and survival by conventional therapeutic agents. In addition to playing a role in proliferation, STAT3 is plays essential roles in cell survival through upregulation of anti-apoptotic molecules such as Bcl-2, Mcl-1, and Survivin. Thus, inhibition of constitutive STAT3 activity by synthetic direct inhibitors or the JAK2 inhibitor AG490 has been shown to induce apoptosis of cancer cells [13,14,21,41-44,48]. However, scoparone exerted no cytotoxic effects on the cancer cell lines we tested, even at the very high concentration of 1 mmol/L, although it caused downregulation of the anti-apoptotic proteins Bcl-2 and Survivin. Nonetheless, scoparone efficiently suppressed the growth of DU145 xenografts in nude mice, accompanied by reduced phosphorylation of STAT3, implying that its anti-tumor efficacy *in vivo* is mediated through inhibition of STAT3. Thus, the cytostatic properties of scoparone suggest the value of this natural compound as a promising anti-cancer agent that could be used in combination therapy with conventional cytotoxic agents. Since the concentration of scoparone required to inhibit proliferation is rather high, more potent analogs of scoparone need to be developed for practical use in cancer treatment.

In summary, we demonstrated that scoparone produces anti-tumor effects against DU145 prostate cancer cells both *in vitro* and *in vivo*. Its action is mediated at least in part by inhibition of STAT3 activity and subsequent repression of transcription of STAT3 target genes. Therefore, our findings suggest that scoparone could represent a novel chemotherapeutic agent against cancers that harbor constitutively active STAT3. Further preclinical studies are needed to investigate the potential for scoparone in combination or adjuvant therapies for the treatment of cancers.

## Supporting Information

Figure S1 **Anti-proliferative effect of scoparone against human hepatoma, a cervical cancer and colon cancer cell lines.** Human hepatocellular carcinoma cell lines (HepG2 and Hep3B), a cervical cancer cell line (HeLa) and colon cancer cell lines (HCT-15, HCT-116 and HT-29) were serum starved for 24 h and incubated in growth medium supplemented with 10% FBS in the presence of vehicle (0.1% DMSO) or the indicated concentrations of scoparone for 72 h. Cell proliferation was determined by WST-8 cell proliferation assay.
(PDF)

Figure S2 **Effect of scoparone on β-catenin and Egr-1-mediated transactivation.** HepG2 cells were transiently cotransfected with pTOPFLASH (A) and Egr-1-Luc (B) reporter constructs together with or without expression plasmids for ΔN-β-catenin (A) or Egr-1 (B), respectively. At 24 h after transfection, cells were treated with scoparone for 24 h, and then harvested for luciferase and β-galactosidase assays. RLU, relative luminescence units. Data are the means ± SEM of three independent experiments, each performed in duplicate. ^*^ *P* < 0.005 vs. reporter alone (A), ^*^ *P* < 0.001 vs. reporter alone, ^**^ *P* < 0.01 vs. Egr-1 (B).
(PDF)

Figure S3 **Effect of scoparone on STAT3 protein level.** Protein levels of STAT3 were quantified by densitometry and normalized against the corresponding levels of β-actin. Expression level of each protein is expressed as a ratio relative to the level in the control at time 0 h (defined as 1). The data represent the means ± S.E.M of three independent experiments.
(PDF)

Figure S4 **Effect of scoparone on protein and mRNA expression of JAK2 and Src.** A and B. Protein levels of JAK2 and Src were quantified by densitometry and normalized against the corresponding levels of β-actin. Expression level of each protein is expressed as a ratio relative to the corresponding level in the control at time 0 h (defined as 1). C. mRNA levels of JAK2 and Src were determined by qRT-PCR analysis and normalized against the level of RPLP0 mRNA. The data represent the means ± S.E.M of three independent experiments, each performed in triplicate.
(PDF)

Table S1 **Primer sequences for qRT-PCR.**
(PDF)

Materials and Methods S1 (DOCX)
