# Supplementary figures and images for: Scoparone Exerts Anti-Tumor Activity against DU145 Prostate Cancer Cells via Inhibition of STAT3 Activity
Source: PLoS One. 2013 Nov 15;8(11):e80391. doi: 10.1371/journal.pone.0080391 (PMC3829856; doi:10.1371/journal.pone.0080391)

Figure S1

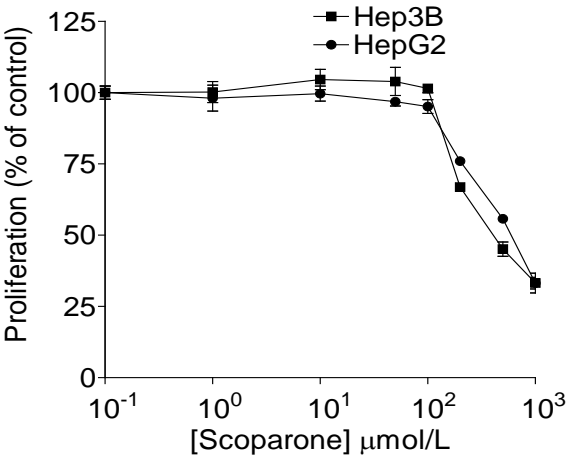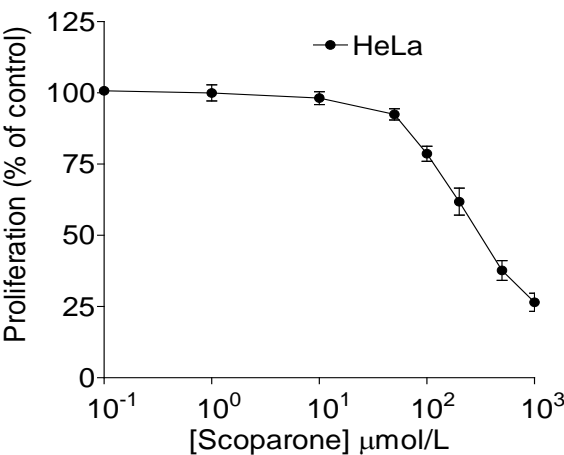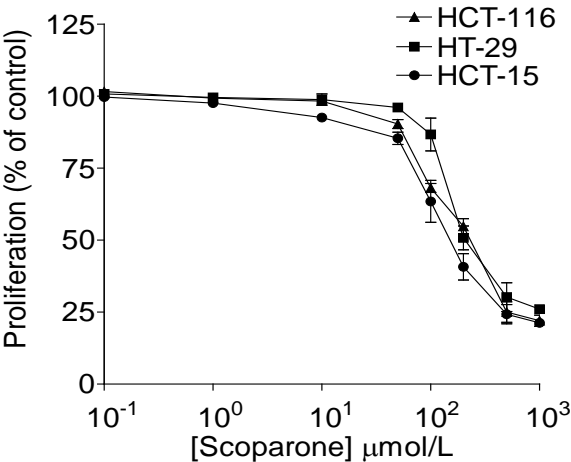

Supplement: Figure S1 — Anti-proliferative effect of scoparone against human hepatoma, a cervical cancer and colon cancer cell lines. Human hepatocellular carcinoma cell lines (HepG2 and Hep3B), a cervical cancer cell line (HeLa) and colon cancer cell lines (HCT-15, HCT-116 and HT-29) were serum starved for 24 h and incubated in growth medium supplemented with 10% FBS in the presence of vehicle (0.1% DMSO) or the indicated concentrations of scoparone for 72 h. Cell proliferation was determined by WST-8 cell proliferation assay. (PDF) [file pone.0080391.s001.pdf]

Figure S2

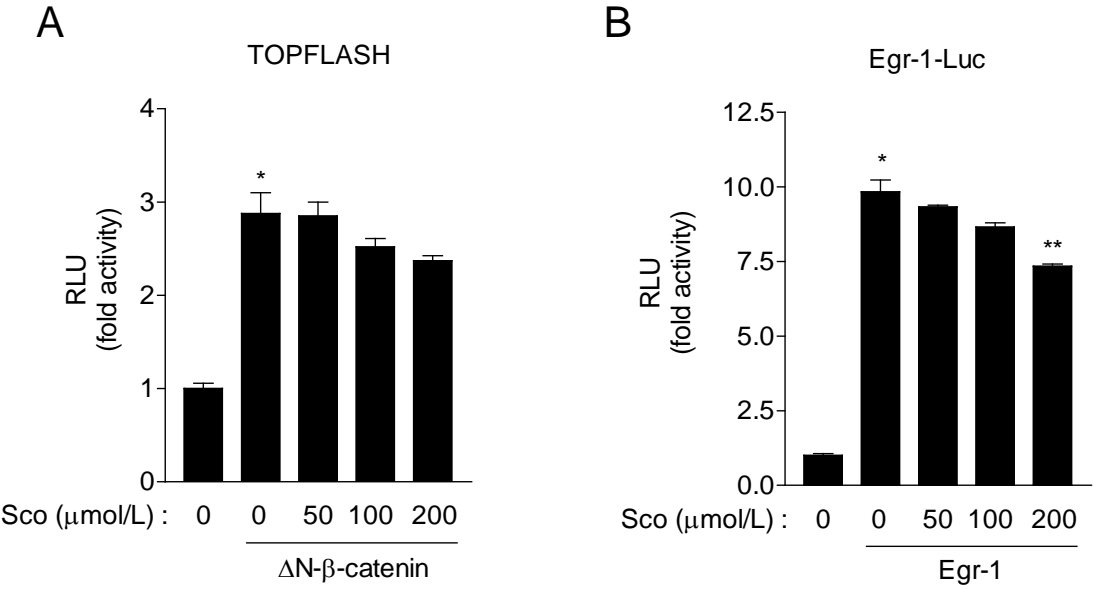

Supplement: Figure S2 — Effect of scoparone on β-catenin and Egr-1-mediated transactivation. HepG2 cells were transiently cotransfected with pTOPFLASH (A) and Egr-1-Luc (B) reporter constructs together with or without expression plasmids for ΔN-β-catenin (A) or Egr-1 (B), respectively. At 24 h after transfection, cells were treated with scoparone for 24 h, and then harvested for luciferase and β-galactosidase assays. RLU, relative luminescence units. Data are the means ± SEM of three independent experiments, each performed in duplicate. * P < 0.005 vs. reporter alone (A), * P < 0.001 vs. reporter alone, ** P < 0.01 vs. Egr-1 (B). (PDF) [file pone.0080391.s002.pdf]

Figure S3

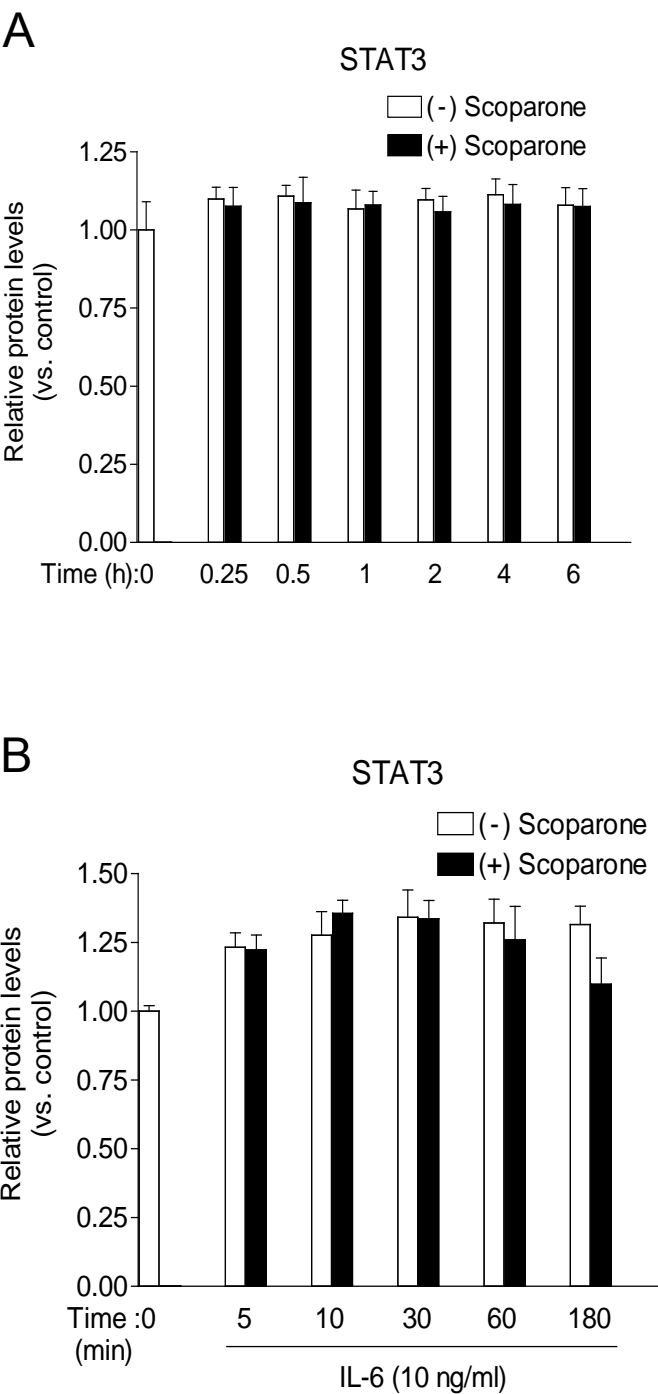

Supplement: Figure S3 — Effect of scoparone on STAT3 protein level. Protein levels of STAT3 were quantified by densitometry and normalized against the corresponding levels of β-actin. Expression level of each protein is expressed as a ratio relative to the level in the control at time 0 h (defined as 1). The data represent the means ± S.E.M of three independent experiments. (PDF) [file pone.0080391.s003.pdf]

Figure S4

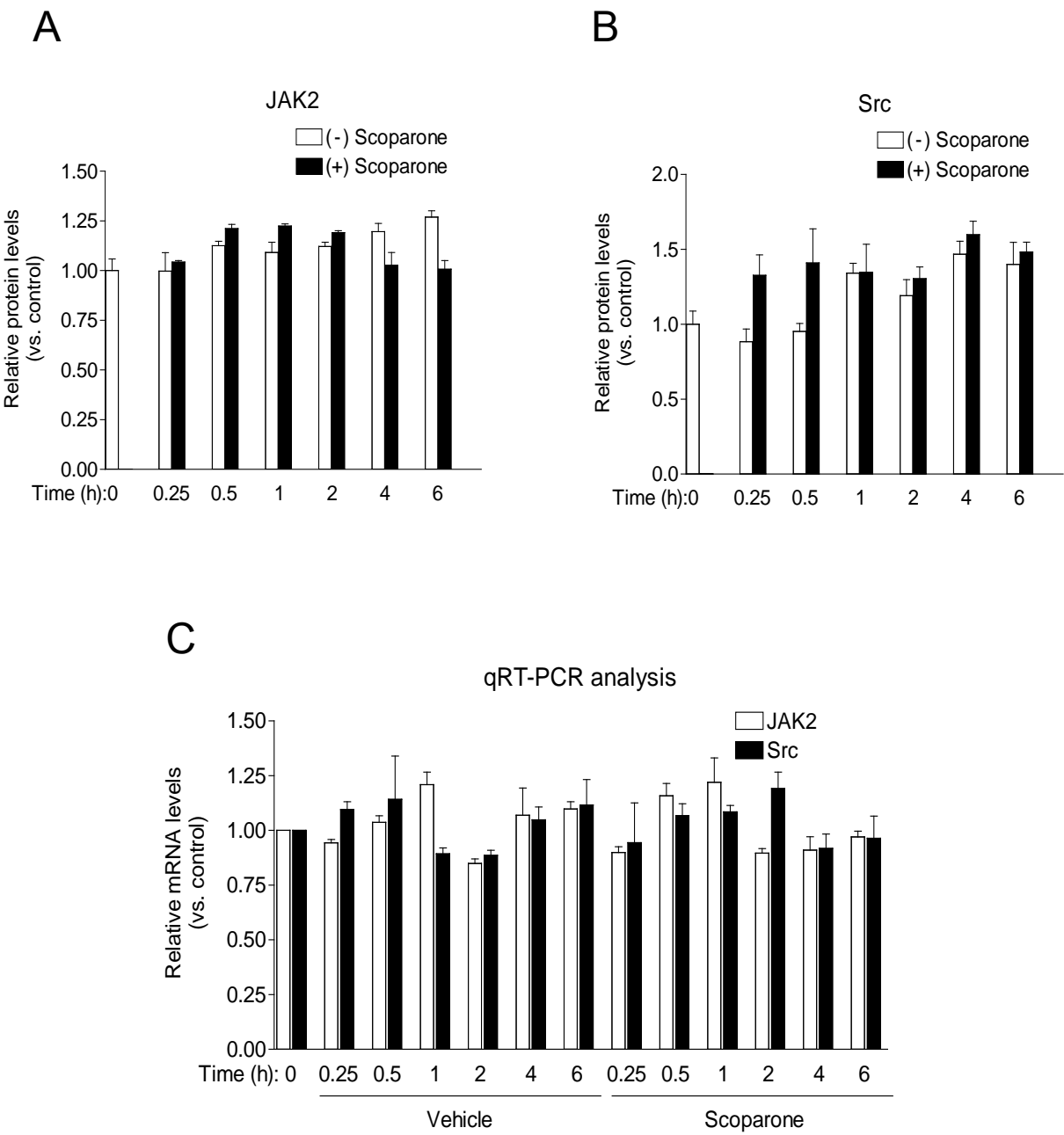

Supplement: Figure S4 — Effect of scoparone on protein and mRNA expression of JAK2 and Src. A and B. Protein levels of JAK2 and Src were quantified by densitometry and normalized against the corresponding levels of β-actin. Expression level of each protein is expressed as a ratio relative to the corresponding level in the control at time 0 h (defined as 1). C. mRNA levels of JAK2 and Src were determined by qRT-PCR analysis and normalized against the level of RPLP0 mRNA. The data represent the means ± S.E.M of three independent experiments, each performed in triplicate. (PDF) [file pone.0080391.s004.pdf]
