# Supplementary material for: Scoparone Exerts Anti-Tumor Activity against DU145 Prostate Cancer Cells via Inhibition of STAT3 Activity
Source: PLoS One. 2013 Nov 15;8(11):e80391. doi: 10.1371/journal.pone.0080391 (PMC3829856; doi:10.1371/journal.pone.0080391)
Supplement: Table S1 — Primer sequences for qRT-PCR. (PDF) [file pone.0080391.s005.pdf]

Table S1. Primer sequences for qRT-PCR

Gene	Primer sequences	Size (bp)	Annealing (°C)	GenBank Accession No.
Cyclin D_1	Forward: 5'- GGTGGCCGCAGTGCAA-3'<br>Reverse: 5'- GAAGCGTGTGAGGCGGTAGTA-3'	72	60	NM_053056
Survivin	Forward: 5'- TCCACTGCCCCACTGAGAAC-3'<br>Reverse: 5'- CAGCCTTCCAGCTCCTTGAA-3'	69	60	NM_001012270
c-Myc	Forward: 5'- GAGGCGAACACACAACGTCTT-3'<br>Reverse: 5'- CACGCAGGGCAAAAAAGC-3'	70	60	NM_002467
BCL2	Forward: 5'- GGGATGCCTTTGTGGAACTG-3'<br>Reverse: 5'- CAGCCAGGAGAAATCAAACAGA-3'	61	60	NM_000633
SOCS3	Forward: 5'- GGACCAGCGCCACTTCTTC-3'<br>Reverse: 5'- ACACTGGATGCGCAGGTTCT-3'	69	60	NM_003955
Jak2	Forward: 5'- TGATTTTGTGCACGGATGGA-3'<br>Reverse: 5'- ACACTGCCATCCCAAGACATTC-3'	73	60	NM_004972
Src	Forward: 5'- GACCTTCGTGCAGCCAACAT-3'<br>Reverse: 5'- CCGAGCCAGCCCAAAGT-3'	71	60	NM_005417
Rplp0/ 36B4	Forward: 5'-CCACGCTGCTGAACATGCT-3'<br>Reverse: 5'-TCGAACACCTGCTGGATGAC-3'	66	60	NM_001002
