# Supplementary material for: Scoparone Exerts Anti-Tumor Activity against DU145 Prostate Cancer Cells via Inhibition of STAT3 Activity
Source: PLoS One. 2013 Nov 15;8(11):e80391. doi: 10.1371/journal.pone.0080391 (PMC3829856; doi:10.1371/journal.pone.0080391)
Supplement: Materials and Methods S1 — (DOCX) [file pone.0080391.s006.docx]

**Supporting Information**

**Supplementary Materials and Methods**

**Constructs**

The Egr-1-Luc construct and expression vector for Egr-1 were described previously (1). The pTOPFLASH luciferase reporter construct (2) and the expression vector for dominant active mutant of human β-catenin (ΔN-β-catenin) containing an in-frame N-terminal deletion of amino acids 29−48 (3) were kindly donated by Dr. Hans Clevers (University Medical Center Utrecht, Utrecht, Netherlands) and Dr. Frank McCormick (University of California, San Francisco, CA, USA), respectively.

**Cell culture and transient transfection**

Human hepatoma cell lines (HepG2 and Hep3B), a cervical cancer cell line (HeLa) and colon cancer cell lines (HT-29, HCT-116 and HCT-15) were obtained from the ATCC. HepG3B and HepG2 cells were grown in DMEM and MEM medium, respectively supplemented with 10% FBS and antibiotics. The other cancer cell lines were grown in RPMI supplemented with 10% FBS and antibiotics. For transient transfection assays, HepG2 (8 × 10^4^) cells were seeded in 24-well plates and transfected with pTOPFLASH or Egr-1-Luc reporter plasmids (200 ng/well) together with or without expression plasmids (100 ng/well) for ΔN-β-catenin or Egr-1, respectively using TransIT-LT1 transfection reagent (Mirus Bio Incorporation, Madison, WI).

**Cell proliferation assay**

Cell proliferation assays were performed using the WST-8 cell proliferation assay kit as previously described (1). Human hepatoma cell lines (HepG2 and Hep3B), a cervical cancer cell line (HeLa) and colon cancer cell lines (HT-29, HCT-116 and HCT-15) were seeded in 96-well plates at a density of 1.5 × 10^3^ cells/well and serum starved for 24 h. Cells were then stimulated with 10% FBS in the presence of 0.1% DMSO (vehicle) or 0.1, 1, 10, 50, 100, 200, 500, or 1000 μmol/L of scoparone for 72 h. Cells were then incubated at 37°C for an additional 2 h in medium containing the WST-8 reagent (Dojindo Laboratories, Kumamoto, Japan). The absorbance at 450 nm was measured to determine cell proliferation.

**References**

1. Kim HJ, Yoo EK, Kim JY, [Choi YK](http://www.ncbi.nlm.nih.gov/pubmed?term=%22Choi%20YK%22%5BAuthor%5D), [Lee HJ](http://www.ncbi.nlm.nih.gov/pubmed?term=%22Lee%20HJ%22%5BAuthor%5D), et al. (2009) Protective role of clusterin/apolipoprotein J against neointimal hyperplasia via antiproliferative effect on vascular smooth muscle cells and cytoprotective effect on endothelial cells. Arterioscler Thromb Vasc Biol 29:1558-64.
2. Tetsu O, McCormick F (1999) Beta-catenin regulates expression of cyclin D1 in colon carcinoma cells. Nature 398:422-6.
3. Kennell JA, O'Leary EE, Gummow BM, Hammer GD, MacDougald OA (2003) T-cell factor 4N (TCF-4N), a novel isoform of mouse TCF-4, synergizes with beta-catenin to coactivate C/EBPalpha and steroidogenic factor 1 transcription factors. Mol Cell Biol 23:5366-75.

**Supplementary Figure Legends**

**Fig. S1.** **Anti-proliferative effect of scoparone against human hepatoma, a cervical cancer and colon cancer cell lines.** Human hepatoma cell lines (HepG2 and Hep3B), a cervical cancer cell line (HeLa) and colon cancer cell lines (HCT-15, HCT-116 and HT-29) were serum starved for 24 h and incubated in growth medium supplemented with 10% FBS in the presence of vehicle (0.1% DMSO) or the indicated concentrations of scoparone for 72 h. Cell proliferation was determined by WST-8 cell proliferation assay.

**Fig. S2.** **Effect of scoparone on β-catenin and Egr-1-mediated transactivation.** HepG2 cells were transiently cotransfected with pTOPFLASH (A) and Egr-1-Luc (B) reporter constructs together with or without expression plasmids for ΔN-β-catenin (A) or Egr-1 (B), respectively. At 24 h after transfection, cells were treated with scoparone for 24 h, and then harvested for luciferase and β-galactosidase assays. RLU, relative luminescence units. Data are the means ± SEM of three independent experiments, each performed in duplicate. ^*^*P* < 0.005 vs. reporter alone (A), ^*^*P* < 0.001 vs. reporter alone, ^**^*P* < 0.01 vs. Egr-1 (B).

**Fig. S3.** **Effect of scoparone on STAT3 protein level.** Protein levels of STAT3 were quantified by densitometry and normalized against the corresponding levels of β-actin. Expression level of each protein is expressed as a ratio relative to the level in the control at time 0 h (defined as 1). The data represent the means ± S.E.M of three independent experiments.

**Fig. S4.** **Effect of scoparone on protein and mRNA expression of JAK2 and Src.** A and B. Protein levels of JAK2 and Src were quantified by densitometry and normalized against the corresponding levels of β-actin. Expression level of each protein is expressed as a ratio relative to the corresponding level in the control at time 0 h (defined as 1). C. mRNA levels of JAK2 and Src were determined by qRT-PCR analysis and normalized against the level of RPLP0 mRNA. The data represent the means ± S.E.M of three independent experiments, each performed in triplicate.

**Supplementary Tables**

**Table S1. Primer sequences for qRT-PCR**

| Gene | Primer sequences | Size (bp) | Annealing (°C) | GenBank Accession |
| --- | --- | --- | --- | --- |
| *Cyclin D1* | Forward: 5’- GGTGGCCGCAGTGCAA-3’ | 72 | 60 | NM_053056 |
|  | Reverse: 5’- GAAGCGTGTGAGGCGGTAGTA-3’ |  |  |  |
| *Survivin* | Forward: 5’- TCCACTGCCCCACTGAGAAC-3’ | 69 | 60 | NM_001012270 |
|  | Reverse: 5’- CAGCCTTCCAGCTCCTTGAA-3’ |  |  |  |
| *c-Myc* | Forward: 5’- GAGGCGAACACACAACGTCTT-3’ | 70 | 60 | NM_002467 |
|  | Reverse: 5’- CACGCAGGGCAAAAAAGC-3’ |  |  |  |
| *BCL2* | Forward: 5’- GGGATGCCTTTGTGGAACTG-3’ | 61 | 60 | NM_000633 |
|  | Reverse: 5’- CAGCCAGGAGAAATCAAACAGA-3’ |  |  |  |
| *SOCS3* | Forward: 5’- GGACCAGCGCCACTTCTTC-3’ | 69 | 60 | NM_003955 |
|  | Reverse: 5’- ACACTGGATGCGCAGGTTCT-3’ |  |  |  |
| *Jak2* | Forward: 5’- TGATTTTGTGCACGGATGGA-3’ | 73 | 60 | NM_004972 |
|  | Reverse: 5’- ACACTGCCATCCCAAGACATTC-3’ |  |  |  |
| *Src* | Forward: 5’- GACCTTCGTGCAGCCAACAT-3’ | 71 | 60 | NM_005417 |
|  | Reverse: 5’- CCGAGCCAGCCCAAAGT-3’ |  |  |  |
| *Rplp0/*  *36B4* | Forward: 5’-CCACGCTGCTGAACATGCT-3’ | 66 | 60 | NM_001002 |
|  | Reverse: 5’-TCGAACACCTGCTGGATGAC-3’ |  |  |  |
